# Cryopreserved Kidney Epithelial (Vero) Cell Monolayers for Rapid Viral Quantification, Enabled by a Combination of Macromolecular Cryoprotectants

**DOI:** 10.1021/acs.biomac.4c00760

**Published:** 2024-07-25

**Authors:** Agnieszka Nagorska, Ruben M. F. Tomás, Afifah Tasnim, Nicole C. Robb, Matthew I. Gibson

**Affiliations:** † CryoLogyx Ltd, Venture Centre, University of Warwick Science Park, Coventry CV4 7EZ, U.K.; ‡ Department of Chemistry, University of Warwick, Coventry CV4 7AL, U.K.; § Division of Biomedical Sciences, Warwick Medical School, University of Warwick, Coventry CV4 7AL, U.K.; ∥ Department of Chemistry, University of Manchester, Oxford Road, Manchester M13 9PL, U.K.; ⊥ Manchester Institute of Biotechnology, University of Manchester, 131 Princess Street, Manchester M1 7DN, U.K.

## Abstract

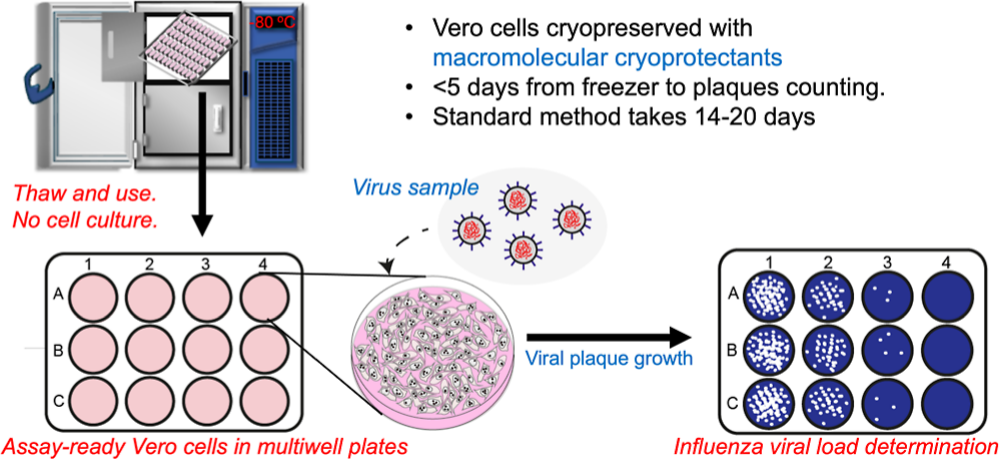

Plaque assays quantify the amount of active, replicating virus to study and detect infectious diseases by application of samples to monolayers of cultured cells. Due to the time taken in thawing, propagating, plating, counting, and then conducting the assay, the process can take over a week to gather data. Here, we introduce assay-ready cryopreserved Vero monolayers in multiwell plates, which can be used directly from the freezer with no cell culture to accelerate the process of plaque determination. Standard dimethyl sulfoxide cryopreservation resulted in just 25% recovery, but addition of polyampholytes (macromolecular cryoprotectants) increased post-thaw recovery and viability in 12- and 24-well plate formats. Variability between individual wells was reduced by chemically induced ice nucleation to prevent supercooling. Cryopreserved cells were used to determine influenza viral plaques in just 24 h, matching results from nonfrozen controls. This innovation may accelerate viral detection and quantification and facilitate automation by eliminating extensive cell culturing.

## Introduction

Quantitative analysis of viral load is crucial for evaluating human viral infections, enabling diagnosis, and determining vaccine safety.^1^ Accurate isolation and quantification of viable viral samples are essential in virology, with plaque assays being the first means to qualitatively and quantitatively measure animal viral titers since 1952.^2^ Although alternative methods for viral quantification exist, such as immunoassays, fluorescence and transmission electron microscopy, tunable resistive pulse sensing (TRPS), flow cytometry, recombinant reporter systems, and quantitative reverse transcription polymerase chain reaction (qRT-PCR), these methods do not identify nor quantify replication-competent virions.^3−5^ Plaque assays remain the gold standard for determining viral concentrations of infectious lytic virions due to their simplicity and relative cost-effectiveness compared to other tools and were an essential tool for measuring infectious SARS-CoV-2 during the COVID-19 pandemic, for example.^6^

In a standard plaque assay, a confluent monolayer of cells is established from frozen (suspension) stocks of a cell line, taking days to weeks to grow depending on the cell line. The virus-containing sample is applied, and individual virions infect cells, replicate, and produce progeny virions, which then infect and kill surrounding cells, creating visible plaques.^7^ Staining, such as with Coomassie blue or crystal violet, is often used to enhance contrast. Vero cells (epithelial kidney cells derived from African green monkeys)^8^ are frequently used for viral particle determination due to their interferon deficiency, making them susceptible to many viruses.^9^ Consequently, they are commonly employed in research laboratories for vaccine development, virus screening, and the growth of viral stocks and vectors.^10,11^ Additionally, they are approved by regulatory authorities, such as the World Health Organization.^12^

A significant barrier in this pipeline is the time required to go from cryopreserved cell stocks in suspension to a confluent monolayer on tissue culture plastic. Cells either need to be cultured for each experiment, which is slow, or continuously cultured, which is resource intensive and risks phenotype drift.^13^ An ideal solution would centralize cell culture, plate the cells in well plates, and cryopreserve them in this preplated format. These cells could be banked long term and used in an “assay-ready” format, thawed, and employed 24 h post-thaw, thereby saving weeks of laboratory time, facilitating screening, and increasing data reproducibility by ensuring phenotypically identical cells and reducing user handling variation.

Direct cryopreservation of cells in an adherent monolayer format presents many challenges. The gold standard cryopreservation solution, 10% dimethyl sulfoxide (DMSO), results in less than 20–40% cell recovery, far below the 80–100% required for plaque assays.^14−16^ The main issues during adherent cell cryopreservation are intracellular ice formation (IIF) caused by insufficient cellular dehydration and detachment from the substrate. IIF is particularly problematic for cryopreserving cells as monolayers compared to suspension cells, as cell–cell contacts promote the propagation of intracellular ice, which is usually fatal and contributes to low cell recovery rates.^17,18^ Insufficient dehydration can be caused by rapid cooling rates,^19^ lack of osmotic gradient, or supercooling. In well plates, supercooling^20^ is more likely to occur, preventing the cryopreservation medium from freezing until approximately −20 °C. Ice formation is an exothermic process, releasing heat during supercooling that can lead to local thawing and affect cooling rates, which hinders cellular dehydration and results in low cell recovery values.^21^ The stochastic nature of ice nucleation causes high variability in freezing and heat of recrystallization across a well plate.

To address the limitations of conventional cryopreservation, macromolecular cryoprotectants have emerged. Polyampholytes, polymers with mixed cationic and anionic side chains, have been shown to promote cellular dehydration by salt entrapment within a matrix surrounding the cells, while minimizing osmotic damage and increasing cell recovery from 50% with DMSO alone to 84%.^15,22^ Polyampholytes also function extracellularly, making them easy to remove. Ice nucleating macromolecules extracted from Hornbeam (*Carpinus betulus*) pollen grains can induce ice nucleation consistently at −7 °C to prevent supercooling, ensuring sufficient dehydration and minimizing well-to-well variability.^23^ This approach has been used for cryopreserving A549, HepG2, and primary hepatocytes in 96-well plates with near-quantitative cells recovered and maintenance of total cell functionality.^24^

In this work, we demonstrate a robust method for cryopreserving Vero cells in monolayer format in both 12- and 24-well plates using polyampholytes combined with chemically-induced ice nucleation. These cells exhibit healthy morphology and cell viability, and grow post-thaw equal to freshly cultured cells. They can be stored at −80 °C and used 24 h post-thaw for plaque assays. Using influenza as a model virus, plaque forming unit (PFU) data obtained from these cryopreserved cells are statistically identical to those from fresh cells, reducing the assay time from 2 to 3 weeks to under 5 days.

## Materials and Methods

### Materials

Vero cells (84113001) were purchased from the European Collection of Authenticated Cell Cultures (ECACC). Poly(methyl vinyl ether-*alt*-maleic anhydride) (*M*_n_ ≈ 80 kDa) (416,339), tetrahydrofuran (THF) (401,757), dimethylamino ethanol (391,263), Eagle’s minimum essential medium (EMEM) with Earle’s salts (M4655), fetal bovine serum (FBS), non-US origin, sterile-filtered (F7524), Dulbecco’s phosphate-buffered saline (DPBS), w/o calcium chloride and magnesium chloride (D8537), trypan blue 0.4% solution (T8154), DMSO Hybri-max, sterile-filtered (D2650), and Corning XT CoolSink 96F were purchased from Merck, Gillingham, UK. Spectrum Laboratories Spectra/Por 5 12–14 kDa MWCO Standard RC Dry Dialysis Kits (15310782); Gibco Antibiotic-Antimycotic (100×) containing penicillin, streptomycin, and amphotericin (PSA) (15240062); Gibco trypsin–EDTA 0.25% (25200072); Invitrogen LIVE/DEAD Viability/Cytotoxicity Kit, for mammalian cells (L3224); and sterile water (15230147) were purchased from Fisher Scientific, Loughborough, UK. Coomassie blue stain (161-0406) was purchased from Bio-Rad, Watford, UK. Carpinus Betulus pollen (hornbeam) was purchased from Pharmallegra, Lišov, Czech Republic.

### Polyampholyte Synthesis

Polyampholyte was synthesized as previously described by Bailey et al.^15,16^ Briefly, poly(methyl vinyl ether-*alt*-maleic anhydride) with an average *M*_n_ value of ≈80 kDa (10 g) was stirred in THF (100 mL), heated to 50 °C, until dissolved. Dimethylamino ethanol (∼10 g) was added in excess, turning the solution from clear to a pink waxy solid. Following 30 min, the solid was dissolved in water (100 mL) and stirred overnight. The THF was removed under vacuum, and the polyampholyte mixture was purified in dialysis tubing (Spectra/Por, 12–14 kDa MWCO). The resulting solution was freeze-dried to form an off-white solid and characterization by ^1^H NMR and FTIR in agreement with previous reports.^16^

### Cell Culture

Epithelial kidney cells derived from African green monkey (Vero) were cultured in EMEM supplemented with 10% FBS and 1% antibiotic–antimycotic solution (PSA). Cells were incubated at 37 °C and 5% CO_2_ and passaged every 3–4 days, before reaching 70–80% confluency, and until a maximum passage number of 20. Cells were passaged using a balanced dissociation salt solution containing trypsin (0.25%) and EDTA (1 mM). Mycoplasma contamination was tested routinely with a MycoAlert Mycoplasma Detection Kit 150 (Lonza, Basel, Switzerland).

### Cryopreservation of Vero Monolayers in 12-Well Plates

Vero cells were seeded in 12-well plates at 50–150 K cells/well for variable confluency and 250 K cells/well for maximum confluency (density selected for experiments, unless specified otherwise) and incubated for 24 h. The medium was aspirated and replaced with 1 mL of base EMEM supplemented with 10% FBS and 10% DMSO or 10% FBS, 10% DMSO, and 40 mg·mL of polyampholyte (0.22 μm sterile filtered). The cells were incubated at RT for 10 min. The freezing solutions were subsequently removed, and the 12-well plates were positioned on a Corning XT CoolSink 96F and placed in a −80 °C freezer overnight. Cells were removed from −80 °C, immediately thawed with warm complete cell media (1 mL, heated to 37 °C), and placed in the incubator for 24 h before conducting viability, biochemical, and virology assays.

### Cryopreservation of Vero Monolayers in 24-Well Plates

Vero cells were seeded in 24-well plates at 25–150 K cells/well for variable confluency and 150 K cells/well for maximum confluency (density selected for experiments, unless specified otherwise) and incubated for 24 h. Optimization of cryopreservation methodology was required, with multiple methods tested. (1) The medium was aspirated and replaced with 500 μL of base EMEM supplemented with 10% FBS and 10% DMSO; 10% FBS, 10% DMSO, and 40 mg·mL of polyampholyte (PA); 10% FBS, 10% DMSO, and 80 mg·mL of polyampholyte (2× PA); or 10% FBS, 10% DMSO, 40 mg·mL of polyampholyte, and 200 mM trehalose (PA + trehalose). The cells were incubated at RT for 10 min. The freezing solutions were subsequently removed, and the 24-well plates were positioned on a Corning XT CoolSink 96F and placed in a −80 °C freezer overnight. Cells were removed from −80 °C, immediately thawed with warm complete cell media (500 μL, heated to 37 °C), and placed in the incubator for 24 h before conducting cell recovery measurements. (2) Polysaccharide ice nucleators were extracted from hornbeam pollen in advance by mixing 0.2 g in 10 mL of sterile water at 4 °C overnight. Pollen was removed via sterile filtration, and the ice nucleator extract solution was mixed 1:1 with base EMEM supplemented with 20% FBS, 20% DMSO, and with (IN + PA) or without (IN) 80 mg·mL of polyampholyte. The medium from the plated cells was aspirated and replaced with 500 μL of the final ice nucleator freezing solution. The cells were incubated at RT for 10 min, and 250 μL of the freezing solution was removed. The 24-well plates were positioned on a Corning XT CoolSink 96F and placed in a −80 °C freezer overnight. Cells were thawed by removing from the −80 °C, adding warm complete cell medium (500 μL, heated to 37 °C), incubating for 12 min (37 °C, 5% CO_2_), and replacing the medium with fresh medium. Cell recovery measurements were completed 24 h post-thaw. Only Vero cells cryopreserved with method 2 were assessed using biochemical and virology assays 24 h post-thaw.

### Calculating Growth Recovery and Growth Rate

Vero cells plated in 12- and 24-well plates were dissociated by using 0.25% trypsin–EDTA (5 min) and stained with 0.02% trypan blue. Viable cells (trypan blue negative) were counted using a hemocytometer immediately before cryopreservation (precount) and 24 h post-thaw to determine the percentage of cells recovered. Percentage cell recovery was calculated by dividing the viable cell count 24 h post-thaw by the precount measurement, multiplied by 100. For growth curves, cells were seeded at a lower density, and cell counts were completed up to 5 days post-thaw, as described previously, to determine proliferation rates.

### Live/Dead Staining

Nonfrozen and freeze/thaw Vero cells, in 12- and 24-well plates, were washed twice with DPBS and incubated with EMEM media containing 2 μM ethidium iodide (EI) and 2 μM calcein at RT for 40 min. The stained cells were imaged by using an Olympus CX41 microscope equipped with a UIS-2 10×/0.45/∞/0–2/FN22 lens. Calcein-positive cells were captured with a 488 nm laser and ethidium iodide-positive cells with a 528 nm laser. Cells were counted using ImageJ’s (v1.52) software cell counter feature. The percentage of live, membrane-intact cells was reported relative to total number of cells.

### Plaque Assays

Nonfrozen Vero cells were seeded at a density of 250,000 cells/well and 150,000 cells/well in 12- and 24-well plates, respectively. The plates were cryopreserved, as described above, thawed with complete EMEM, and incubated for 24 h to allow cell recovery. The cell culture medium was removed, and the cells were washed twice with DPBS. A stock solution of influenza virus (strain A/WSN/33 (H1N1)) was propagated in Madin–Darby Canine Kidney (MDCK) cells before being serially diluted 1:10 in EMEM media containing 0.5% FBS and added to the VERO cells (200 μL) for 1 h. The viral solution was aspirated, and a 1% agarose overlay was added, prepared by mixing a 2% melted agarose solution in PBS 1:1 with MEM media containing 0.5% FBS. Once the overlay solidified, plates were incubated at 37 °C until visible plaques formed. The overlay was removed after 96 h, and cells were stained with Coomassie blue for 1 h. Visible plaques were imaged, and PFU were quantified by multiplying the number of plaques by 10 and the serial dilution factor.

## Results and Discussion

**Figure 1:**
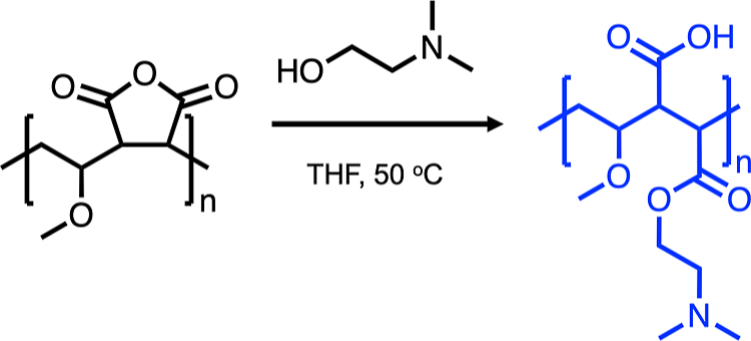
Cryoprotecting polyampholyte synthesis.

Our primary objective was to cryopreserve Vero cells as 2-D monolayers, enabling their immediate use for viral screening assays directly from the freezer without additional processing steps, thereby eliminating the challenges associated with routine cell culture. Viral infectivity studies, such as plaque assays, require cells to be 80–100% confluent, necessitating total cell recovery post-thaw, which underscores the significant technical challenge of this process. DMSO, the gold standard cryoprotectant, is insufficient to achieve high recovery and viability for preplated cells. Consequently, we selected a macromolecular cryoprotectant previously utilized for cell monolayer cryopreservation (Figure 1).^15^ This cryoprotectant was synthesized through the ring-opening polymerization of poly(methyl vinyl ether-*alt*-maleic anhydride) with dimethylamino ethanol, ensuring a precise 1:1 balance of cationic and anionic groups by the anhydride ring opening. This method maximizes cryopreservation benefits and avoids the challenges associated with the copolymerization of two distinct monomers, which can result in uneven monomer distribution due to reactivity ratio differences.^25,26^

The efficiency of polyampholyte in cryopreserving Vero cells adhered to 12- and 24-well plates was evaluated by replacing the cell culture medium with a cryopreservation solution containing 40 mg·mL^–1^ of polyampholyte (PA) dissolved in 10% FBS, 10% DMSO, and EMEM. A control without polyampholyte was also used for comparison. The cells were incubated with the cryoprotectant at room temperature for 10 min before excess cryoprotectant was removed and then placed directly in a −80 °C freezer for cryopreservation. After 24 h, the cells were thawed with complete cell culture medium and incubated for an additional 24 h. This post-thaw recovery period is essential to eliminate false positives associated with short post-thaw measurements, which can overestimate recovery by not allowing sufficient time for apoptosis to occur.^27^

**Figure 2:**
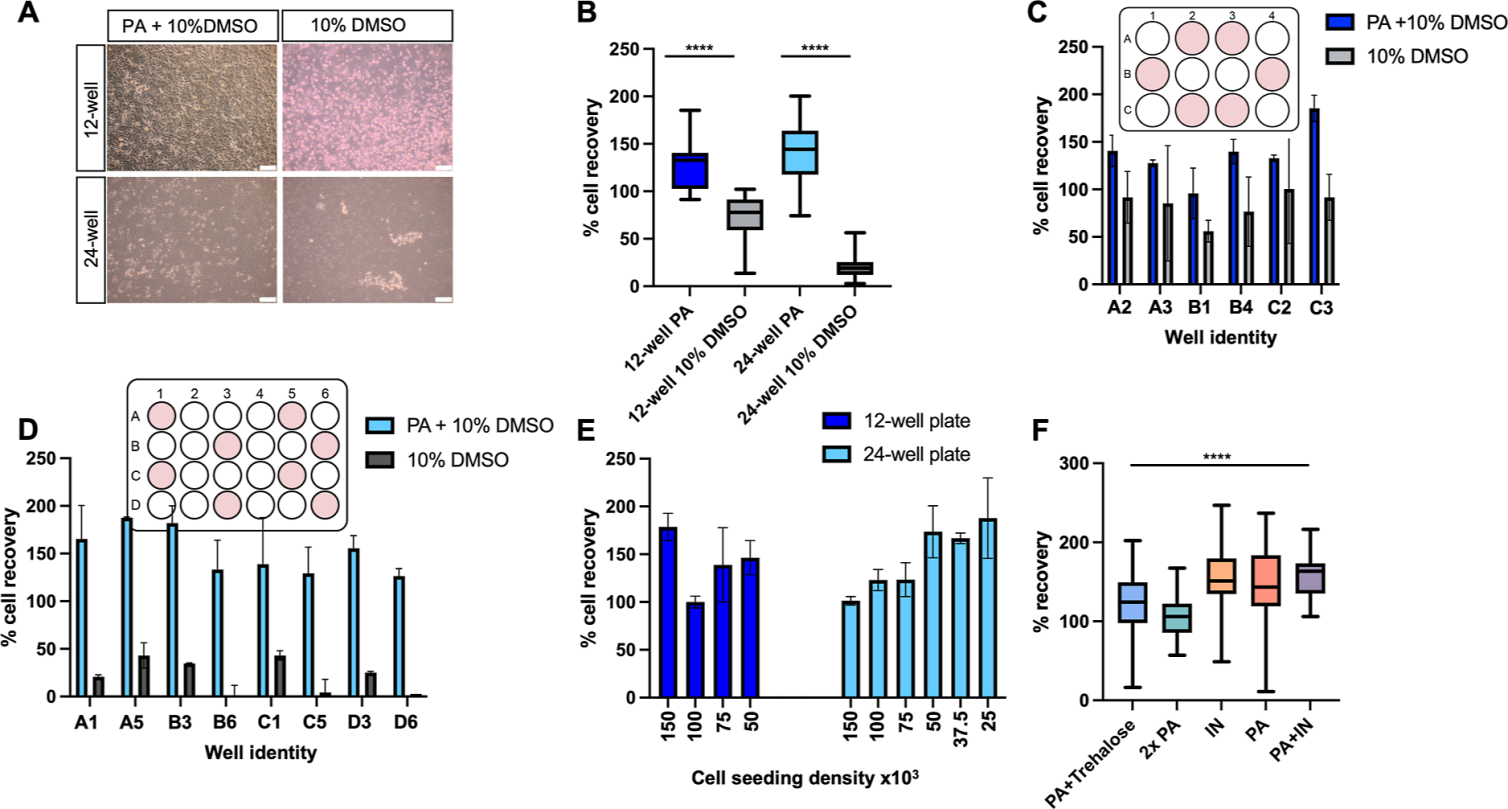
Post-thaw recovery of Vero monolayers cryopreserved in 12- and 24-well plates. (A) Microscopy images of recovered cells. Scale bar = 100 μm; (B) cell recovery 24 h post-thaw, following cryopreservation in 12- and 24-well plates. [Polyampholyte] = 40 mg·mL^–1^, DMSO = 10%. Statistical analysis: Student’s *t*-test, *P* value < 0.0001; (C) individual well location recovery values for a 12-well plate; (D) individual well location recovery values for a 24-well plate; (E) cell recovery following cryopreservation at different cell seeding densities in 12- and 24-well plates; (F) cell recovery following cryopreservation in a 24-well plate with 40 mg·mL^–1^ polyampholyte and 200 μM trehalose (PA + trehalose), 80 mg·mL^–1^ polyampholyte (2× PA), ice nucleator only (IN), and 40 mg·mL^–1^ polyampholyte and ice nucleator (PA + IN). Statistical analysis was completed using a one-way ANOVA, *P* value < 0.0001.

Microscopy images collected post-thaw revealed that Vero cells cryopreserved using a 10% DMSO solution detached in both the 12- and 24-well microplate formats (Figure 2A). In contrast, the addition of polyampholyte resulted in significantly more attached cells. Post-thaw cell recovery was quantified by comparing cell counts immediately before freezing and 24 h post-thaw. Vero monolayers cryopreserved with 10% DMSO in 12-well plates had an average cell recovery of 70%, while those in 24-well plates had only 25% cell recovery (Figure 2B). However, when polyampholyte was added, cell recovery dramatically increased to 120 and 140%, respectively. Vero cells cryopreserved with polyampholyte were capable of proliferating within the first 24 h post-thaw, as reflected in the >100% cell recovery, confirming normal cell functionality immediately post-thaw. Most reports on Vero cells involve storage in suspension (cryovials) with a slow rate of freezing, making data comparison difficult. However, it has been shown that some polysaccharides, when added to 96-well plate Vero monolayers, can increase post-thaw recoveries by 2–3-fold compared to the 10% DMSO control.^28^ Although promising, virology applications require confluent Vero monolayers, which, to the best of our knowledge, have not been previously reported. These initial results highlight the limitations of using 10% DMSO for monolayer cryopreservation and underscore the need for innovative cryoprotectants to cryopreserve cells in preplated formats. The mechanism of protection of polyampholytes is still under investigation, but there is evidence they help dehydrate the cell^29^ (reducing the extent of intracellular ice formation) or modulate ion transport.^30^ They also have weak ice recrystallization inhibition (IRI) activity,^31^ which is unlikely to be a major contributor to the results seen here, compared to the extent of benefit for more active IRIs.^32^ Percentage cell recovery was also calculated for multiple wells within a single plate to determine possible well-to-well variability (Figure 2C,D), which could critically impact the reproducibility of the data obtained by end-users. No significant “hot or cold spots” were identified among the wells tested. Additionally, different Vero cell seeding densities were cryopreserved to assess their impact on post-thaw cell recovery. Although not crucial for virology applications, which require 80–100% confluent cells, different cell densities may be necessary for assays where a linear relationship between cell seeding density and signal output is required, such as WST-8 and MTT assays. At any given seeding density, the percentage cell recovery remained around 100% (Figure 2E), confirming compatibility with various assays.

To further improve Vero monolayer cryopreservation in 24-well plates and minimize the possibility of well-to-well variability, various formulation additives were tested (Figure 2F). Doubling the concentration of polyampholyte to 80 mg·mL^–1^ showed no improvements in the total or well-to-well range of cells recovered, which ranged from 57 to 167%. Adding trehalose (200 μM), a common nonpermeating cryoprotectant with benefits in monolayer cryopreservation,^33,34^ also showed no benefits, with percentage cell recovery ranging from 24 to 171%.

Poor post-thaw outcomes are associated with small volumes (<100 μL) during cryopreservation which increases the likelihood of water supercooling. In the event of supercooling, ice nucleation is delayed to temperatures as low as −20 °C, leading to increased osmotic stress, intracellular ice formation, and cell detachment.^21,35^ The stochastic nature of ice nucleation also means that supercooling can occur randomly among wells, so each well freezes at a different temperature, increasing well-to-well variability and lowering reproducibility within and between plates. To address this, a fully water-soluble ice nucleating macromolecule extracted from pollen grains, which has recently enabled 96-well plate monolayer cryopreservation, was added to the cryopreservation formulation to increase the nucleation temperature and protect cells during cryopreservation.^23,36^ Controls of the ice nucleator with 10% DMSO and 10% FBS and polyampholyte with 10% DMSO and 10% FBS were also employed for comparison. Interestingly, we found that cryopreserved cells with either induced nucleation or polyampholyte solution produced comparable results, with percentage cell recovery post-thaw ranging from 49 to 218% for ice nucleator only and 50 to 189% with 40 mg·mL^–1^ polyampholyte included. This supports our hypothesis that polyampholytes promote cellular dehydration, which is also an effect of induced ice nucleation.^23,29^ Combining polyampholyte with induced ice nucleation together significantly improved cell recovery and reduced variability between samples, with percentage cell recovery ranging from 106.12 to 212.12% (Figure 2F).

Both polyampholyte and induced nucleation reduce deleterious intracellular ice growth by promoting dehydration, albeit through different mechanisms,^29,30^ explaining the similarities in results when used independently. Evidence suggests that polyampholyte protection is due to salt entrapment by a matrix surrounding the cells, minimizing osmotic damage while ensuring sufficient dehydration to prevent spontaneous intracellular ice formation. Additionally, polyampholytes may interact with and protect the cell membrane,^37^ similar to how antifreeze proteins protect.^38^

**Figure 3:**
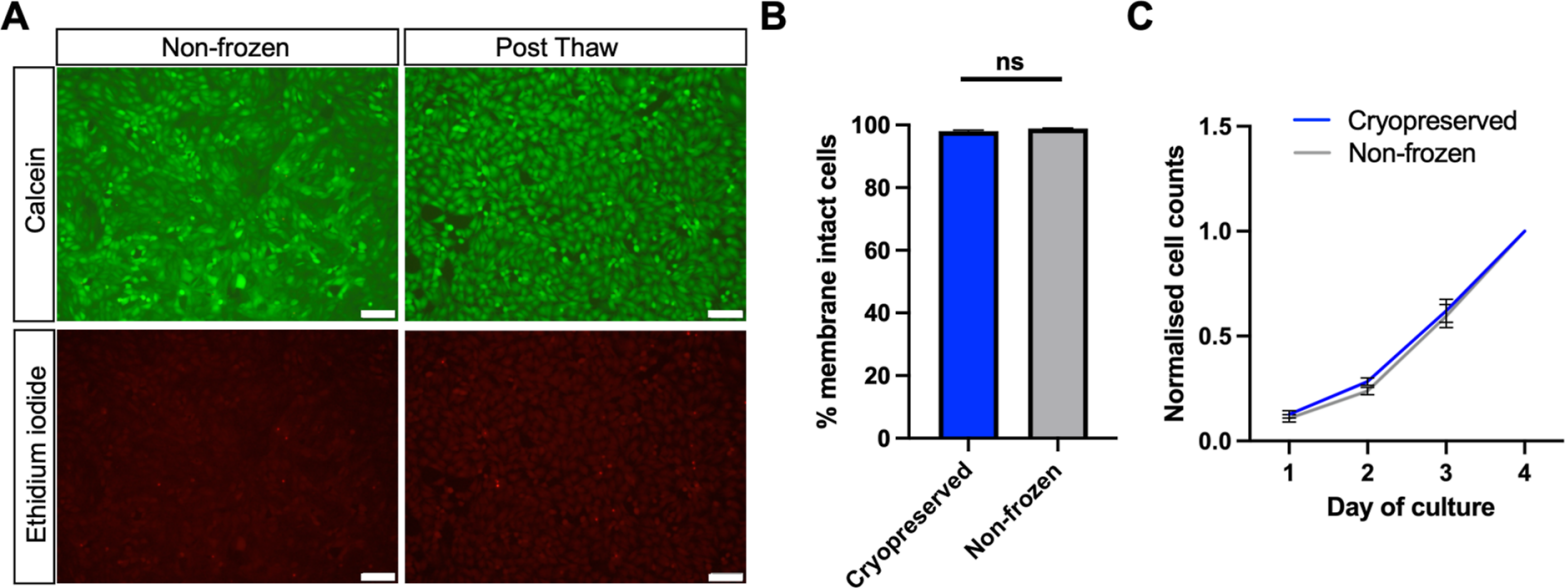
Vero cells show normal morphology and proliferation 24 h post-thaw. (A) Fluorescent images of nonfrozen and freeze/thaw Vero monolayers stained with calcein (green, membrane intact) and ethidium iodide (red, membrane damage). Scale bar = 100 μm; (B) proportion of membrane-intact cells of freeze/thaw and nonfrozen Vero monolayers calculated from live/dead images; and (C) normalized growth curve of freeze/thaw and nonfrozen Vero cells over 4 days of culturing. Cryopreservation solution contained 10% DMSO, 40 mg·mL^–1^ polyampholyte, and ice nucleating solution.

Live/dead staining (calcein and ethidium iodide) on both nonfrozen and frozen (using both polyampholyte and nucleation) Vero cells was used to further assess cell viability. Cells stained green with calcein indicate healthy, membrane-intact cells, while red-stained cells with ethidium iodide indicate cells with damaged membranes. Freeze/thawed Vero cells showed 98% membrane-intact cells 24 h post-thaw, which were identical to the fresh cells (Figure 3A,B). The growth rate of the thawed cells was subsequently assessed by cell counting over a 5 day period (Figure 3C). Thawed and nonfrozen Vero cells had doubling times of 24 and 23 h, respectively, leading to the conclusion that these macromolecular cryoprotectants enable the recovery of viable, healthy, and functional cells.

**Figure 4:**
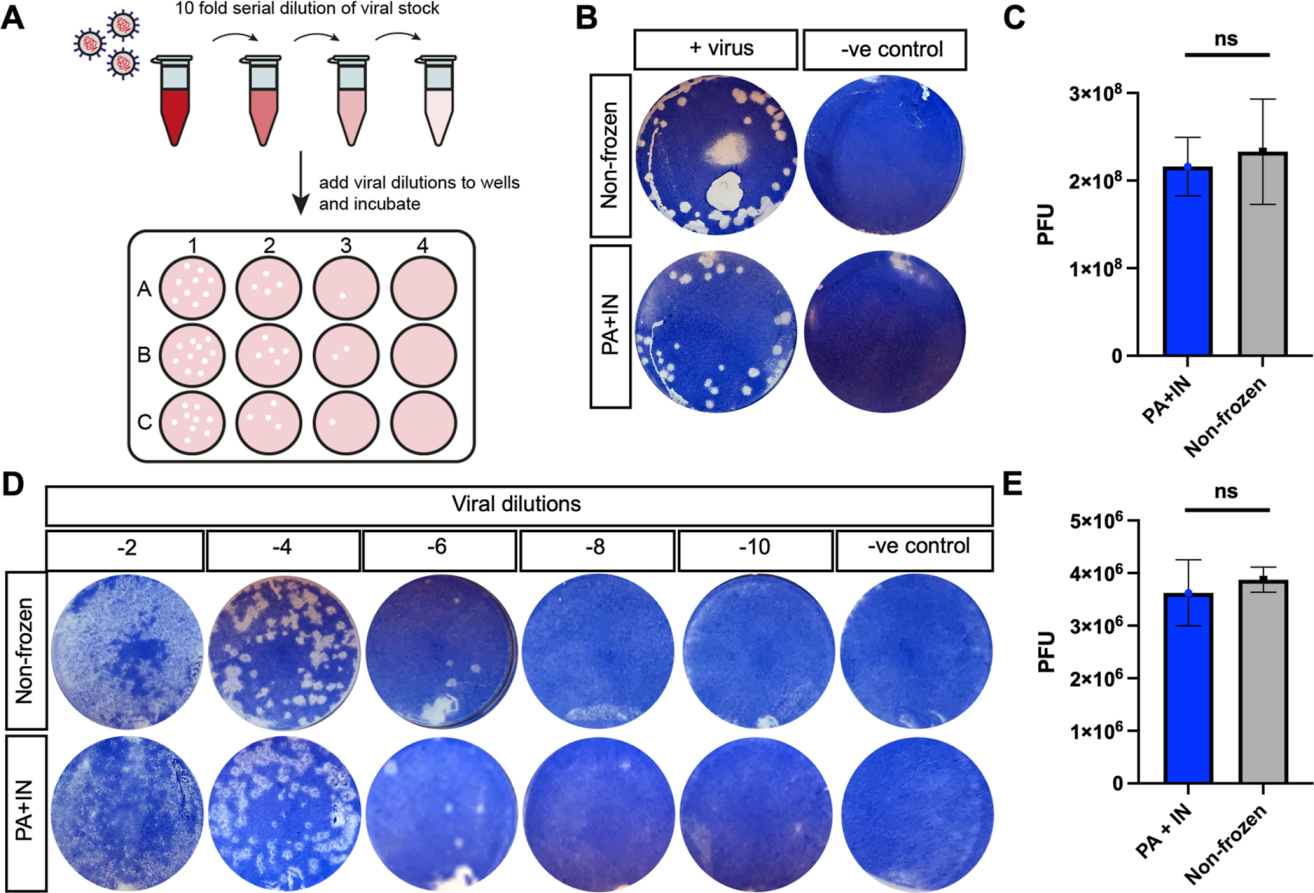
Plaque assays performed using cryopreserved Vero 12- and 24-well plates. (A) Schematic of plaque assay protocol; (B) images of nonfrozen and freeze/thawed cells, in 12-well plates, incubated with (+virus) or without (−ve control) 10^–6^ viral dilution of H1N1 and stained with Coomassie blue; and (C) quantification of PFUs in the 12-well plates. Student’s *t*-test *P* value not significant. (D) Images of nonfrozen and freeze/thaw cells, in 24-well plates, incubated with 10^–2^ to 10^–10^ viral dilutions of H1N1 and stained with Coomassie blue; (E) quantification of PFUs for 24-well plate, Student’s *t*-test *P* value not significant. Cryopreservation solution contained 10% DMSO, 40 mg·mL^–1^ polyampholyte, and ice nucleating solution.

Vero cells are widely used for virology applications due to their deficiencies in interferon expression, which promotes susceptibility to many viruses.^10^ Plaque assays are particularly useful for studying viral infectivity and determining viable virus counts, such as for SARS-CoV-2.^39^ An advantage of preplated cryopreserved Vero cells in well plates is the ability to accelerate viral screening by simply removing the plates from the freezer, eliminating the need for cell culture and reducing the time, effort, and consumables required for the assays. Vero cells cryopreserved in 12- and 24-well plates using the optimized polyampholyte with induced nucleation solution were thawed and incubated for 24 h. A dilution series of influenza virus A/WSN/33 (H1N1) was then added to the plates. After 4 days, viral plaques were quantified and compared to the nonfrozen controls (Figure 4A).

Figure 4 shows the viral plaque counting results after 4 days of incubation in both 12- and 24-well plate formats, demonstrating that the cryopreserved plates yielded results nearly identical to those of fresh cells. In the 12-well format, frozen plates had PFUs of 2.16 × 10^8^, compared to 2.33 × 10^8^ for nonfrozen Vero cells (Figure 4B,C). For the 24-well plates, the PFU values were 3.88 × 10^6^ for frozen plates and 3.63 × 10^6^ for the nonfrozen controls (Figure 4D,E). This confirms that preplated cryopreserved Vero cells perform identically to routinely cultured cells in a major functional viral challenge. Comparing the cryopreserved method to conventional culture, the time taken from thawing either a vial or a plate to obtaining data was 3 weeks and 5 days, respectively. This represents a significant time saving and optimization enabled by the unique cryoprotectants deployed here.

## Conclusions

Herein, we demonstrate that Vero cells can be cryopreserved preplated on well plates and used directly from a −80 °C freezer in influenza viral plaque counting assays. This method eliminates the need for tedious cell culture, reducing the time required to obtain data from 3 weeks to 5 days. Cryopreservation was achieved using a combination of emerging macromolecular cryoprotectants as conventional DMSO-alone cryopreservation resulted in less than 30% cell recovery and high variability between individual wells, which would compromise assay performance. A synthetic polyampholyte significantly increased cell recovery, consistent with previous reports on other cell lines. Chemically induced ice nucleation, using macromolecules isolated from pollen, was supplemented into the polyampholyte solution to minimize well-to-well variability. The combination of polyampholyte and ice nucleators provided a synergistic effect, offering near-quantitative cell recovery, minimal variability, normal proliferation rates, and stable confluent cell monolayers post-thaw. Viral plaque assays conducted on the cryopreserved Vero cells with influenza strain A/WSN/33 (H1N1) showed near-identical PFU results compared with freshly cultured cells. Overall, this study demonstrates a new approach to enable rapid and high-throughput viral testing with minimal processing and effort facilitated by innovative cryopreservation technology using macromolecular cryoprotectants.

## References

[ref1] Clementi M. Quantitative Molecular Analysis of Virus Expression and Replication. J. Clin. Microbiol. 2000, 38 (6), 2030–2036. 10.1128/JCM.38.6.2030-2036.2000. 10834949 PMC86721

[ref2] Dulbecco R. Production of Plaques in Monolayer Tissue Cultures by Single Particles of an Animal Virus. Proc. Natl. Acad. Sci. U.S.A. 1952, 38 (8), 747–752. 10.1073/pnas.38.8.747. 16589172 PMC1063645

[ref3] Baer A.; Kehn-Hall K. Viral Concentration Determination through Plaque Assays: Using Traditional and Novel Overlay Systems. J. Vis. Exp. 2014, 93, e52065 10.3791/52065. PMC4255882 25407402

[ref4] Masci A. L.; Menesale E. B.; Chen W. C.; Co C.; Lu X.; Bergelson S. Integration of Fluorescence Detection and Image-Based Automated Counting Increases Speed, Sensitivity, and Robustness of Plaque Assays. Mol. Ther.--Methods Clin. Dev. 2019, 14, 270–274. 10.1016/j.omtm.2019.07.007. 31489337 PMC6717064

[ref5] Liu T.; Li Y.; Koydemir H. C.; Zhang Y.; Yang E.; Eryilmaz M.; Wang H.; Li J.; Bai B.; Ma G.; et al. Rapid and Stain-Free Quantification of Viral Plaque via Lens-Free Holography and Deep Learning. Nat. Biomed. Eng. 2023, 7 (8), 1040–1052. 10.1038/s41551-023-01057-7. 37349390 PMC10427422

[ref6] Mendoza E. J.; Manguiat K.; Wood H.; Drebot M. Two Detailed Plaque Assay Protocols for the Quantification of Infectious SARS-CoV-2. Curr. Protoc. Microbiol. 2020, 57 (1), ecpmc105 10.1002/cpmc.105. 32475066 PMC7300432

[ref7] Payne S. Methods to Study Viruses. Viruses; Elsevier, 2017; pp 37–52.

[ref8] Nahapetian A. T.; Thomas J. N.; Thilly W. G. Optimization of Environment for High Density Vero Cell Culture: Effect of Dissolved Oxygen and Nutrient Supply on Cell Growth and Changes in Metabolites. J. Cell Sci. 1986, 81 (1), 65–103. 10.1242/jcs.81.1.65. 3733899

[ref9] Kiesslich S.; Kamen A. A. Vero Cell Upstream Bioprocess Development for the Production of Viral Vectors and Vaccines. Biotechnol. Adv. 2020, 44, 107608 10.1016/j.biotechadv.2020.107608. 32768520 PMC7405825

[ref10] Sène M.; Xia Y.; Kamen A. A. Overview of Recent Advances in Vero Cells Genomic Characterization and Engineering for High-Throughput Vaccine Manufacturing. Clin. Transl. Discovery 2022, 2 (2), e40 10.1002/ctd2.40.

[ref11] Trabelsi K.; Rourou S.; Loukil H.; Majoul S.; Kallel H. Optimization of Virus Yield as a Strategy to Improve Rabies Vaccine Production by Vero Cells in a Bioreactor. J. Biotechnol. 2006, 121 (2), 261–271. 10.1016/j.jbiotec.2005.07.018. 16153733

[ref12] Shen C. F.; Guilbault C.; Li X.; Elahi S. M.; Ansorge S.; Kamen A.; Gilbert R. Development of Suspension Adapted Vero Cell Culture Process Technology for Production of Viral Vaccines. Vaccine 2019, 37 (47), 6996–7002. 10.1016/j.vaccine.2019.07.003. 31288997

[ref13] Torsvik A.; Stieber D.; Enger P. O.; Golebiewska A.; Molven A.; Svendsen A.; Westermark B.; Niclou S. P.; Olsen T. K.; Chekenya Enger M.; et al. U-251 Revisited: Genetic Drift and Phenotypic Consequences of Long-Term Cultures of Glioblastoma Cells. Cancer Med. 2014, 3 (4), 812–824. 10.1002/cam4.219. 24810477 PMC4303149

[ref14] Pless-Petig G.; Knoop S.; Rauen U. Serum- and Albumin-Free Cryopreservation of Endothelial Monolayers with a New Solution. Organogenesis 2018, 14 (2), 107–121. 10.1080/15476278.2018.1501136. 30081735 PMC6150062

[ref15] Bailey T. L.; Stubbs C.; Murray K.; Tomas R. M. F.; Otten L.; Gibson M. I. Synthetically Scalable Poly(ampholyte) Which Dramatically Enhances Cellular Cryopreservation. Biomacromolecules 2019, 20, 3104–3114. 10.1021/acs.biomac.9b00681. 31268698 PMC6692820

[ref16] Tomás R. M. F.; Bissoyi A.; Congdon T. R.; Gibson M. I. Assay-Ready Cryopreserved Cell Monolayers Enabled by Macromolecular Cryoprotectants. Biomacromolecules 2022, 23, 3948–3959. 10.1021/acs.biomac.2c00791. 35972897 PMC9472225

[ref17] Acker J. P.; McGann L. E. Cell-Cell Contact Affects Membrane Integrity after Intracellular Freezing. Cryobiology 2000, 40 (1), 54–63. 10.1006/cryo.1999.2221. 10679150

[ref18] Acker J. P.; Elliott J. A. W.; McGann L. E. Intercellular Ice Propagation: Experimental Evidence for Ice Growth through Membrane Pores. Biophys. J. 2001, 81 (3), 1389–1397. 10.1016/S0006-3495(01)75794-3. 11509353 PMC1301618

[ref19] Mazur P.; Leibo S. P.; Chu E. H. Y. A two-factor hypothesis of freezing injury. Exp. Cell Res. 1972, 71 (2), 345–355. 10.1016/0014-4827(72)90303-5. 5045639

[ref20] Bigg E. K. The Supercooling of Water. Proc. Phys. Soc., London, Sect. B 1953, 66 (8), 688–694. 10.1088/0370-1301/66/8/309.

[ref21] Daily M. I.; Whale T. F.; Partanen R.; Harrison A. D.; Kilbride P.; Lamb S.; Morris G. J.; Picton H. M.; Murray B. J. Cryopreservation of Primary Cultures of Mammalian Somatic Cells in 96-Well Plates Benefits from Control of Ice Nucleation. Cryobiology 2020, 93, 62–69. 10.1016/j.cryobiol.2020.02.008. 32092295 PMC7191264

[ref22] Matsumura K.; Hyon S. H. Polyampholytes as Low Toxic Efficient Cryoprotective Agents with Antifreeze Protein Properties. Biomaterials 2009, 30 (27), 4842–4849. 10.1016/j.biomaterials.2009.05.025. 19515417

[ref23] Murray K. A.; Kinney N. L. H.; Griffiths C. A.; Hasan M.; Gibson M. I.; Whale T. F. Pollen Derived Macromolecules Serve as a New Class of Ice-Nucleating Cryoprotectants. Sci. Rep. 2022, 12 (1), 12295 10.1038/s41598-022-15545-4. 35854036 PMC9296471

[ref24] Tomás R. M. F.; Dallman R.; Congdon T. R.; Gibson M. I. Cryopreservation of Assay-Ready Hepatocyte Monolayers by Chemically-Induced Ice Nucleation: Preservation of Hepatic Function and Hepatotoxicity Screening Capabilities. Biomater. Sci. 2023, 11 (23), 7639–7654. 10.1039/D3BM01046E. 37840476 PMC10661096

[ref25] Zhao J.; Johnson M. A.; Fisher R.; Burke N. A. D.; Stöver H. D. H. Synthetic Polyampholytes as Macromolecular Cryoprotective Agents. Langmuir 2019, 35 (5), 1807–1817. 10.1021/acs.langmuir.8b01602. 30134094

[ref26] Stubbs C.; Murray K. A.; Ishibe T.; Mathers R. T.; Gibson M. I. Combinatorial Biomaterials Discovery Strategy to Identify New Macromolecular Cryoprotectants. ACS Macro Lett. 2020, 9, 290–294. 10.1021/acsmacrolett.0c00044. 32337092 PMC7175595

[ref27] Murray K. A.; Gibson M. I. Post-Thaw Culture and Measurement of Total Cell Recovery Is Crucial in the Evaluation of New Macromolecular Cryoprotectants. Biomacromolecules 2020, 21 (7), 2864–2873. 10.1021/acs.biomac.0c00591. 32501710 PMC7362331

[ref28] Guerreiro B. M.; Concórdio-Reis P.; Pericão H.; Martins F.; Moppert X.; Guézennec J.; Lima J. C.; Silva J. C.; Freitas F. Elevated Fucose Content Enhances the Cryoprotective Performance of Anionic Polysaccharides. Int. J. Biol. Macromol. 2024, 261, 129577 10.1016/j.ijbiomac.2024.129577. 38246459

[ref29] Bissoyi A.; Tomás R. M. F.; Gao Y.; Guo Q.; Gibson M. I. Cryopreservation of Liver-Cell Spheroids with Macromolecular Cryoprotectants. ACS Appl. Mater. Interfaces 2023, 15 (2), 2630–2638. 10.1021/acsami.2c18288. 36621888 PMC9869333

[ref30] Matsumura K.; Hayashi F.; Nagashima T.; Rajan R.; Hyon S.-H. Molecular Mechanisms of Cell Cryopreservation with Polyampholytes Studied by Solid-State NMR. Commun. Mater. 2021, 2 (1), 15 10.1038/s43246-021-00118-1.

[ref31] Stubbs C.; Lipecki J.; Gibson M. I. Regioregular Alternating Polyampholytes Have Enhanced Biomimetic Ice Recrystallization Activity Compared to Random Copolymers and the Role of Side Chain versus Main Chain Hydrophobicity. Biomacromolecules 2017, 18 (1), 295–302. 10.1021/acs.biomac.6b01691. 27936601 PMC5271573

[ref32] Bailey T. L.; Hernandez-Fernaud J. R.; Gibson M. I. Proline Pre-Conditioning of Cell Monolayers Increases Post-Thaw Recovery and Viability by Distinct Mechanisms to Other Osmolytes. RSC Med. Chem. 2021, 12 (6), 982–993. 10.1039/d1md00078k. 34223163 PMC8221256

[ref33] Stokich B.; Osgood Q.; Grimm D.; Moorthy S.; Chakraborty N.; Menze M. A. Cryopreservation of Hepatocyte (HepG2) Cell Monolayers: Impact of Trehalose. Cryobiology 2014, 69 (2), 281–290. 10.1016/j.cryobiol.2014.08.001. 25127872

[ref34] Bailey T. L.; Wang M.; Solocinski J.; Nathan B. P.; Chakraborty N.; Menze M. A. Protective Effects of Osmolytes in Cryopreserving Adherent Neuroblastoma (Neuro-2a) Cells. Cryobiology 2015, 71 (3), 472–480. 10.1016/j.cryobiol.2015.08.015. 26408850

[ref35] Daily M. I.; Whale T. F.; Kilbride P.; Lamb S.; John Morris G.; Picton H. M.; Murray B. J. A Highly Active Mineral-Based Ice Nucleating Agent Supports in Situ Cell Cryopreservation in a High Throughput Format. J. R. Soc., Interface 2023, 20 (199), 20220682 10.1098/rsif.2022.0682. 36751925 PMC9905984

[ref36] Murray K. A.; Gao Y.; Griffiths C. A.; Kinney N. L. H.; Guo Q.; Gibson M. I.; Whale T. F. Chemically Induced Extracellular Ice Nucleation Reduces Intracellular Ice Formation Enabling 2D and 3D Cellular Cryopreservation. JACS Au 2023, 3 (5), 1314–1320. 10.1021/jacsau.3c00056. 37234117 PMC10207112

[ref37] Rajan R.; Hayashi F.; Nagashima T.; Matsumura K. Toward a Molecular Understanding of the Mechanism of Cryopreservation by Polyampholytes: Cell Membrane Interactions and Hydrophobicity. Biomacromolecules 2016, 17 (5), 1882–1893. 10.1021/acs.biomac.6b00343. 27077533

[ref38] Sun Y.; Maltseva D.; Liu J.; Hooker T.; Mailänder V.; Ramløv H.; DeVries A. L.; Bonn M.; Meister K. Ice Recrystallization Inhibition Is Insufficient to Explain Cryopreservation Abilities of Antifreeze Proteins. Biomacromolecules 2022, 23 (3), 1214–1220. 10.1021/acs.biomac.1c01477. 35080878 PMC8924859

[ref39] Sasaki M.; Uemura K.; Sato A.; Toba S.; Sanaki T.; Maenaka K.; Hall W. W.; Orba Y.; Sawa H. SARS-CoV-2 Variants with Mutations at the S1/S2 Cleavage Site Are Generated in Vitro during Propagation in TMPRSS2-Deficient Cells. PLoS Pathog. 2021, 17 (1), e1009233 10.1371/journal.ppat.1009233. 33476327 PMC7853460

